# Gas-assisted microfluidic step-emulsification for generating micron- and submicron-sized droplets

**DOI:** 10.1038/s41378-023-00558-4

**Published:** 2023-07-10

**Authors:** Biao Huang, Xinjin Ge, Boris Y. Rubinstein, Xianchun Chen, Lu Wang, Huiying Xie, Alexander M. Leshansky, Zhenzhen Li

**Affiliations:** 1 Department of Aerospace Engineering, Beijing Institute of Technology, No. 5 ZhongGuanCunNan Street, HaiDian District, Beijing, 100081 China; 2 State Key Laboratory of Engines, Tianjin University, No. 92 Weijin Road, Nankai District, Tianjin, 300350 China; 3 Stowers Institute for Medical Research, Kansas City, MO 64110 USA; 4 School of Chemistry and Chemical Engineering, Beijing Institute of Technology, No. 5 ZhongGuanCunNan Street, HaiDian District, Beijing, 100081 China; 5 Department of Chemical Engineering, Technion – Israel Institute of Technology, Haifa, 32000 Israel

**Keywords:** Nanofluidics, Nanoparticles

## Abstract

Micron- and submicron-sized droplets have extensive applications in biomedical diagnosis and drug delivery. Moreover, accurate high-throughput analysis requires a uniform droplet size distribution and high production rates. Although the previously reported microfluidic coflow step-emulsification method can be used to generate highly monodispersed droplets, the droplet diameter (*d*) is constrained by the microchannel height (*b*), $d\gtrsim 3b$, while the production rate is limited by the maximum capillary number of the step-emulsification regime, impeding emulsification of highly viscous liquids. In this paper, we report a novel, gas-assisted coflow step-emulsification method, where air serves as the innermost phase of a precursor hollow-core air/oil/water emulsion. Air gradually diffuses out, producing oil droplets. The size of the hollow-core droplets and the ultrathin oil layer thickness both follow the scaling laws of triphasic step-emulsification. The minimal droplet size attains $d\approx 1.7b$, inaccessible in standard all-liquid biphasic step-emulsification. The production rate per single channel is an order-of-magnitude higher than that in the standard all-liquid biphasic step-emulsification and is also superior to alternative emulsification methods. Due to low gas viscosity, the method can also be used to generate micron- and submicron-sized droplets of high-viscosity fluids, while the inert nature of the auxiliary gas offers high versatility.

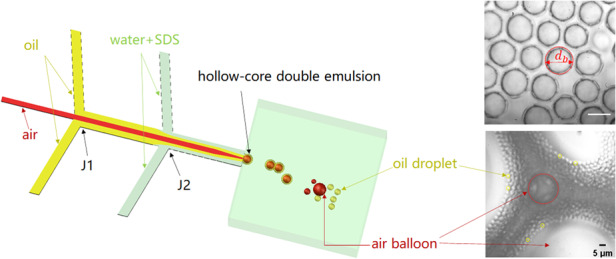

## Introduction

Droplets and particles of micron and submicron size are widely used in targeted drug delivery and controlled release^1^, and the submicron size facilitates their transport in the intracellular environment and their endocytosis by the cells^2^. Similarly, bubbles and perfluorocarbon droplets are used as contrast agents in ultrasound detection and therapy^3,4^. Depending on the application, the chemical composition of particles or droplets varies from solutes to macromolecules, such as proteins, polymers, and DNA^1^. Likewise, the viscosity of the disperse phase fluid ranges from 1 cP for aqueous biofluids, including blood plasma and serum^5^, to 1000 cP or higher for concentrated polymer solutions. Existing methods for making nanodroplets and nanoparticles include high-pressure homogenization and sonication^1^, low-energy methods such as self-emulsification, which rely on the extraction of solvent from the dispersed phase to the continuous phase to shrink the droplets to the submicron scale, and the phase inversion temperature or component (PIT or PIC), which involves the inversion of curvature of the interface at a critical temperature or by the addition of an auxiliary chemical component^6^. However, the existing methods are selective in the chemical composition of the phases, and it is challenging to form droplets with a uniform size distribution. The latter problem can be solved by using microfluidic techniques, where the droplet size is tightly controlled by the geometry of the T-junction microchannel^7^, Y-junction microchannel^8^, flow focusing or coflow method^9^. In these techniques, the droplet size is regulated by the microchannel dimension^10,11^, and, therefore, generating submicron-sized droplets requires microchannels of submicron width and height, which is technically challenging. For example, Toprakcioglu et al. reported a T-junction nanochannel geometry to generate nanodroplets of controllable size^7^. Other methods were proposed to downscale the droplet size. For instance, small capillaries assembled into PDMS channels can generate droplets and bubbles whose sizes are controlled by the capillary inner diameter, and such hybrid devices are realized in a flow-focusing geometry^4^.

Tip-streaming is an effective way to generate monodispersed micron- and submicron-sized droplets when the interface is subjected to shear or elongation flow that drags the surfactants at the interface toward the tip of the disperse phase stream, resulting in a considerable reduction in the local surface tension. Then, a thin thread is drawn out of the stagnant tip, breaking up the stream into tiny droplets^9^. A robust tip-streaming method can be realized in a hybrid device with a flame-shaped capillary assembled with a PDMS channel that allows for 3D focusing of the dispersed thread^4^. A similar device was used for emulsification of water as well as highly viscous polyethylene glycol at the submicron scale^11^. Tip streaming provides good conditions for the formation of micron and submicron droplets; however, it is restricted to a specific range of surfactant concentrations and to low-viscosity ratio fluids, i.e., ${\mu }_{d}/{\mu }_{c}\, < \,{\mathscr{O}}(0.1)$, where ${\mu }_{d}$ and ${\mu }_{c}$ are the viscosities of the disperse and continuous phases, respectively^12^.

Emulsification of high-viscosity fluids is challenging when using standard microfluidic methods since the interfacial deformation required for subsequent thread breakup is suppressed by viscosity^13^. Alternative strategies have been proposed; for instance, low-viscosity droplets can be first generated in a high-viscosity continuous phase by flow focusing before the system undergoes a phase inversion by switching the surface wettability to form high-viscosity droplets suspended in a low-viscosity continuous phase^14^.

Microfluidic step-emulsification (SE) is an emerging technology used for the generation of highly monodisperse microdroplets at high throughput^15–17^. The dispersed phase does not wet the channel walls, and a convex interface with the continuous phase (wetting the walls) is formed, resulting in an extra Laplace (capillary) pressure within the dispersed phase thread. Theory based on quasistatic equilibrium explains the reason for thread necking as the curvature in the thread balancing the curvature of the growing drop^18^. An alternative theory of coflow step-emulsification considers that the competition of viscous and surface tension forces can be used to compute the interfacial profile based on Hele–Shaw hydrodynamics^19–21^. Experimentally, improvements in the local geometry near the step were proposed, such as the addition of a constriction and bypass channel^22,23^ and a shunt channel that facilitates adverse flow^24^ and promotes thread pinching, increasing the droplet production rate. When the dispersed fluid flowing in the Hele-Shaw channel partially wetted the walls (i.e., different from the coflow SE where the dispersed phase does not wet the channel walls), it was found that the contact angle of the dispersed fluid forming with the channel plays an essential role in determining the droplet size^25^. The viscous shear effect has a marginal effect on the droplet size, rendering it insensitive to fluctuating flow rates, while it is mainly controlled by the Hele-Shaw channel height, *b*^26^. To generate micron- and submicron-sized droplets produced with the coflow step-emulsification technique, ultrashallow channels with heights <1 µm could be used^26,27^, which require sophisticated nanofabrication and high operating pressures due to high hydraulic resistance. The triphasic coflow SE was recently proposed to generate double emulsions with ultrathin shells of thickness ~1.6% of the droplet diameter^28^.

Hollow-core structures can be generated in a microfluidic device by encapsulating gas bubbles within liquid drops. A gas-core triple emulsion was shown to be able to release encapsulated fluid triggered by ultrasound^29^. Single bubbles in a solution may shrink due to the dissolution of the gas into the unsaturated liquid phase^30^. Microbubbles suspended in lipid solution can dissolve into the aqueous phase to form nanobubbles that can be used as ultrasound contrast agents (UCAs)^31^. A mixture of perfluorocarbon (PFC) with another component that is soluble in the continuous phase can be emulsified into drops^32^ or bubbles^33^ and subsequently reduced to smaller-sized PFC. This idea is similar to methods based on solvent evaporation or extraction^34^.

Droplet generation methods involving gas were previously explored in various microfluidic geometries; for example, in a T-junction device, bubbles were used to trigger pinching of the thread of the disperse phase^35^. Another method used laser excitation to create a bubble near the free surface on the liquid side, and bubble expansion caused a shock wave to propagate toward the interface, triggering a jet of liquid droplets^36^. Air can also be used as the focusing fluid (i.e., continuous phase), which drives liquid thread breakup and droplet formation^37^. To the best of our knowledge, the generation of micron- and submicron-sized droplets (especially at high disperse-to-continuous viscosity ratios) remains a challenging undertaking for conventional microfluidic methods with passive flow control.

In the present work, we propose a gas-assisted coflow step-emulsification (SE) method to first generate precursor hollow-core double emulsions, which subsequently produce small, single emulsions of the disperse phase upon dissolution of the encapsulated gas cores. Smaller micron-sized droplets at higher throughput can be generated compared with the standard biphasic SE methods. Therefore, single emulsions of micron- and submicron-sized droplets can be readily generated using the auxiliary coflow of air.

## Results and discussion

### Mechanism of droplet formation

#### Triphasic coflow step-emulsification microfluidic device

**Fig. 1:**
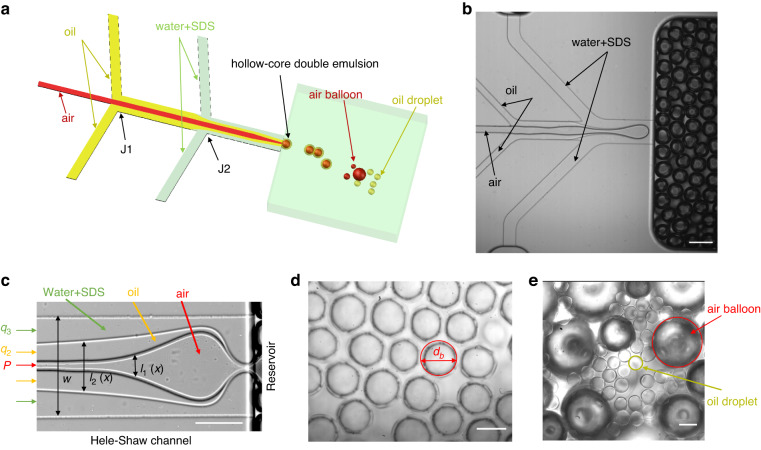
Oil droplets formation by air-assisted step-emulsification. **a** Schematic diagram of the device for producing hollow-core double emulsions. The air, oil (FC40) and SDS aqueous solutions are the inner disperse, outer disperse and continuous phases, respectively. The air meets the oil phase at the first junction (J1), and the air-oil compound threads meet the aqueous phase at the second junction (J2). Hollow-core double emulsions are formed at the step. Further downstream in the reservoir, the air cores shrink and disappear or diffuse into air “balloon” bubbles, leaving oil single-emulsion droplets. **b** Top view experimental image of the microfluidic device in (**a**). **c** Close-up top view experimental image of the interfaces separating the three fluid phases in the Hele-Shaw channel. **d** Self-assembled hollow-core double emulsions in the reservoir close to the step. **e** A mixture of oil single drops and large air balloons downstream in the reservoir. Scale bar lengths are 200 μm in (**b**), 100 μm in (**c**) and (**d**), and 50 μm in (**e**)

As sketched in Fig. 1a and the experimental picture in Fig. 1b, the device possesses three inlets for the injection of three phases, with air being the innermost disperse phase, FC40 oil being the outer disperse phase, and the SDS aqueous solution being the continuous phase. The air thread is enclosed by the oil when they meet at the first junction (J1), and this compound thread is enclosed by the continuous aqueous phase at the second junction (J2). The three phases are confined and coflow through the Hele-Shaw channel with a high aspect ratio $\beta =w/b \,>\, 10$ before arriving at the step, where the Hele-Shaw channel enters a deep and wide reservoir. Here, *b* and *w* are the Hele-Shaw channel height and width, respectively. It should be noted that the air flow velocity in the Hele-Shaw channel is on the order of 10 cm/s, so the effects of compressibility can be safely neglected, as the Mach number is well below 0.3. In this situation, the pressure contributes to the flow of the air instead of compressing the air. Figure 1c shows a close-up top image of the interfaces between the three phases in the Hele-Shaw channel, and Supplementary Figure S1a schematically shows the structure of the compound thread in a cross-section of the Hele-Shaw cell.

We observe that the compound (containing the air and oil phases) disperse phase thread breaks synchronously at the step, forming hollow-core double emulsions (Supplementary Movie S1 and Fig. 1b), which pack into an ordered array (Fig. 1d) in the reservoir due to the low velocity of the carrier aqueous phase. The step-emulsification process is stable for hours (Supplementary Movie S2). These hollow-core double emulsions are thermodynamically stable, as the spreading parameters satisfy ${S}_{3}={\gamma }_{12}-\left({\gamma }_{13}+{\gamma }_{23}\right)\,<\,{0}$ and ${S}_{2}={\gamma }_{13}-\left({\gamma }_{12}+{\gamma }_{23}\right)\,>\,{0}$^38^, where ${\gamma }_{12}=16.09\,$ mN/m is the surface tension between oil (FC40) and air, ${\gamma }_{23}=15.62$ mN/m is that between oil (FC40) and SDS aqueous solution, and ${\gamma }_{13}=33.63$ mN/m is that between air and the SDS solution. Downstream in the reservoir, near the outlet to the atmosphere, the air cores shrink and disappear on a timescale on the order of seconds, as shown in Supplementary Movie S3, leaving a single oil-in-water emulsion (Fig. 1e). The shrinkage of the air cores is caused by diffusion of the air through the oil phase and dissolution in the unsaturated aqueous phase^30^. In addition, Ostwald ripening, which corresponds to air diffusing from a smaller bubble to a larger bubble driven by the difference in the Laplace pressure, is apparently operative as the mechanism of the bubble shrinkage^39^. It can be readily seen in Fig. 1e that a large number of small oil droplets are formed around large air balloons. The large air balloons are readily eliminated after being exposed to ambient atmosphere, terminating their mission in gas-assisted generation of FC40 droplet emulsions. As shown in Supplementary Movie S3, the diffusion of air from individual hollow-core double emulsion droplets does not result in their coalescence. At the exit of the channel, the evaporation of the continuous phase at ambient atmosphere and temperature is not significant, so a stable oil-in-water emulsion can be obtained. The shrunken FC40 droplets have diameters as low as 1.7*b*. Previous research on biphasic coflow step-emulsification theoretically predicted that the diameter of the droplet cannot be smaller than ~3*b*^19^. In this work, the gas-assisted step-emulsification of the oil droplet overcomes this theoretical limitation. We note that during the generation of hollow-core double emulsions, the shell encapsulating the innermost air bubble can be made ultrathin by tuning the oil flowrate, so the oil shell cannot be detected using an optical microscope. However, based on the diameter of the final droplet that can be measured, the thickness of the shell of the precursor hollow-core double emulsions can be easily estimated. In the following subsections, we study various dynamic regimes of droplet formation depending on the relevant dimensionless parameters and investigate the variation in the droplet size and generation rate.

#### Dynamic regimes of emulsification: phase diagram

**Fig. 2:**
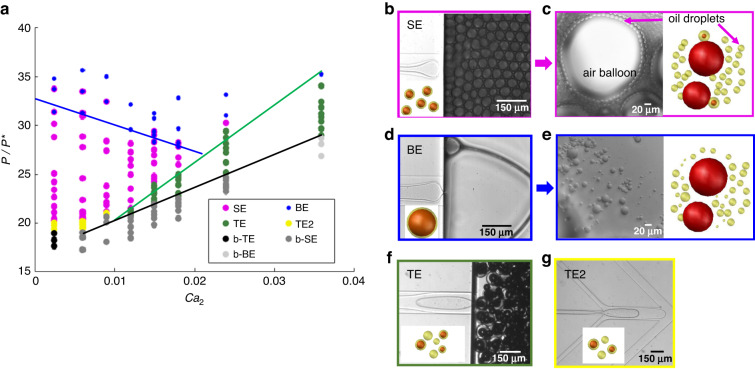
Regimes of droplet formation. **a** Phase diagram of double hollow-core emulsification in the plane of dimensionless air pressure $\hat{P}=P/{P}^{* }$ and capillary number of the oil, ${{Ca}}_{2}$. The Hele-Shaw channel has a width $w=150\,\upmu \rm{m}$ and a height $b=5\,\upmu \rm{m}$. The aqueous (continuous) phase flow rate ${q}_{3}$ = 100 $\upmu \rm{L/h}$. Seven emulsification regimes are observed: SE shown in (**b**), and the subsequent oil droplets with uniform size distribution with diameter around $2b$ following the air core shrinkage, observed downstream of the reservoir, shown in (**c**); BE shown in (**d**), and the oil droplets with size ranging from submicrons to tens of microns observed in the reservoir shown in (**e**), the T-junction emulsification (TE) as shown in (**f**), and the double T-junction emulsification (TE2) shown in (**g**). Black, dark gray and light gray dots in (**a**) correspond to biphasic emulsion formations, and the corresponding micrographs are shown in Supplementary Fig. S2. Blue, green and black solid lines in (**a**) showing the boundaries among different regimes are for eye guidance

We controlled the pressure of the inner dispersed air (*P*) and the flowrates of the outer dispersed FC40 oil (${q}_{2}$) and of the continuous aqueous SDS solution (${q}_{3}$). Seven different regimes of droplet formation are observed depending on the governing parameters, as shown in the phase diagram in Fig. 2a depicted in the plane of the dimensionless inlet air pressure $\hat{P}=P/{P}^{* }$ and capillary number of the oil phase, ${{Ca}}_{2}=12{\mu }_{2}{q}_{2}/{\gamma }_{23}{bw}$. Here, *P* is the air pressure; ${P}^{* }={\gamma }_{12}/b$ is the characteristic capillary (Laplace) pressure in the Hele-Shaw channel; ${\mu }_{2}$ and ${q}_{2}$ are the viscosity and the flow rate of the outer disperse (oil) phase, respectively; ${\gamma }_{23}$ is the surface tension between the oil and the aqueous phases; and *b* and *w* are the Hele-Shaw channel height and width, respectively.

Among the seven regimes in Fig. 2a, there are four regimes that yield the formation of hollow-core double emulsions, which are of interest to this study. The first regime, designated “T-junction emulsification (TE)” (green dots in Fig. 2a and f), consists of the case when the air bubble is formed at the first junction (J1), and the bubble-laden oil thread enters the Hele-Shaw cell and undergoes emulsification at the step. This yields the formation of a mixture of single oil emulsions and hollow-core double emulsions with thick oil shells. As a consequence, it is challenging to maintain a uniform distribution of the droplet diameter and the shell thickness, as shown in Fig. 2f. In a similar work by Opalski et al.^40^, single droplets were first generated in a flow focusing in a channel, and the resulting emulsion entered another channel, with fine control of the flow rate, yielding the formation of a double emulsion.

Gradually increasing the air inlet pressure, the TE regime bifurcates into two additional regimes: the “step-emulsification (SE)” regime at smaller ${q}_{2}$, where the compound (oil-air) thread breaks at the step (magenta dots in Fig. 2a and b), generating highly monodisperse hollow-core double emulsions at high frequency. Oil droplets at a uniform distribution with a diameter of approximately 2*b* are formed downstream of the step following air core shrinkage, as shown in Fig. 2c; and the “balloon-emulsification (BE)” regime at higher ${q}_{2}$, where large hollow-core droplets (“balloons”) are formed at low frequency (blue dots in Fig. 2a and d), droplets with a wide size distribution ranging from submicron to tens of microns are produced downstream of the step (Fig. 2e). Submicron droplets are rarely formed by air bubbles shrinking from a hollow-core double emulsion, and they probably originate from oil film dewetting. The dewetting process was recorded using silicon oil with a viscosity of 69 mPa.s, as shown in Supplementary Movie S4. It can be seen that a number of holes in the oil film were formed on the bubble surface during bubble growth at the step. We speculate that the fast rupture is caused by the van der Waals forces (disjoining pressure) that come into play for an ultrathin oil film^41^.

In the phase diagram (Fig. 2a), the SE regime is bounded from above by the BE regime, and this boundary is in the form of a linear function with a negative slope (see blue line). This is in qualitative agreement with the prediction by Ge et al.^28^ In the case of triphasic coflow liquid step-emulsification, ${{Ca}}_{1}+\gamma {{Ca}}_{2}\, < \,\gamma {\overline{{Ca}}}_{2* }$, with ${{Ca}}_{1}$ and ${{Ca}}_{2}$ being the capillary numbers of the inner and outer disperse phases, respectively, which are proportional to their corresponding flow rates; ${\overline{{Ca}}}_{2* }$ is a critical value of the effective capillary number ${\overline{{Ca}}}_{2}=\frac{12({q}_{1}+{q}_{2}){\widetilde{\mu }}_{12}}{{\gamma }_{23}{bw}}$, which accounts for the flow rate of both disperse phases. Here, ${\widetilde{\mu }}_{12}=\frac{{\mu }_{1}{q}_{1}+{\mu }_{2}{q}_{2}}{{q}_{1}+{q}_{2}}$ is the flux-averaged viscosity and $\gamma ={\gamma }_{23}/{\gamma }_{12}$ is the ratio of surface tension coefficients. Since in our work the air flow is pressure-controlled, a quantitative comparison with the theory^28^ is not readily accessible. Converting the air pressure into the air flow rate ${q}_{1}$ for all regimes is technically challenging. However, in the next section (i.e., study on droplet size in the SE regime), we estimate ${q}_{1}$ from experimental measurements of the droplet size and production rate, which allows for quantitative comparison with the theory. On the other hand, another boundary separating the TE regime from the SE and BE regimes drawn as the green solid line in Fig. 2a has physical meaning that the thread width of the inner disperse phase equals the channel height, so that below this boundary (at smaller pressure of the air), the thread breaks at the first junction (J1) to form bubbles due to Rayleigh-Plateau instability, as predicted by Ge et al.^28^. Hence, the phase diagram of the air-assisted step-emulsification is in qualitative agreement with the theory developed for the triphasic liquid step-emulsification.

Starting at low values of ${q}_{2}$, corresponding to the SE regime and gradually decreasing inlet air pressure, another regime emerges, where the air thread breaks at the first junction (J1) and the oil thread breaks at the second junction (J2), forming a compound droplet of oil laden within a confined bubble. This drop breaks at the step (yellow dots in Fig. 2a and g) forming a mixture of hollow-core double emulsions and single oil droplets. This regime is called “double T-junction emulsification (TE2)”. A similar method relying on a single “slug” droplet breaking into N identical droplets at the step was used for the fabrication of colloidal clusters of predesigned geometry^42^. However, the droplet production rate in this regime is relatively low. Moreover, this regime is restricted to a very narrow range of parameters, limiting its practical applicability for generating hollow-core double emulsions.

At low pressure (below the critical pressure, which increases with increasing ${{Ca}}_{2}$, as shown by the black line in Fig. 2a), the air “finger” cannot reach the first junction (J1), so only the oil thread can be emulsified at the step. Upon increasing the flowrate ${q}_{2}$, the transition occurs from a biphasic T-junction emulsification (b-TE), whereas the oil thread breaks at the second junction, to biphasic step-emulsification (b-SE) and biphasic balloon-emulsification (b-BE) regimes, designated in Fig. 1a as black, dark gray and light gray dots, respectively (the images are shown in Supplementary Fig. S2).

In the next subsection, we shall discuss the droplet size and production rate in the SE regime followed by bubble shrinkage and dissolution. This experimental method is validated by using different oils, such as FC40, phenylmethyl silicon oil and mineral oil, as shown in Supplementary Figure S3, demonstrating the wide chemical compatibility of the method. Supplementary Figure S4a shows a phase diagram for phenylmethyl silicon oil replacing silicon oil as an alternative outer dispersed phase; the fluid properties used for the phase diagrams (Fig. 2 and Fig. S4) are compared in Supplementary Table S1. The similarity between the two diagrams demonstrates the broad versatility of the method. When compared to the phase diagram in Fig. 2a, the locations of various emulsification regimes in Fig. S4a are shifted along both parameter axes (${\hat{P},{Ca}}_{2}$), as the position and/or slopes of the boundaries separating them depend on the surface tension ratio $\gamma$ (see Ge et al.^28^), which is different in the two cases (see Supplementary Table S1).

#### Droplet size and production rate

In this section, we focus on the SE regime and analyze the size and shell thickness of the hollow-core double emulsions. We fix the flow rate of the continuous phase at ${q}_{3}=100$ $\mu L/h$ and modify the flow rate of the outer disperse phase, ${q}_{2}$, and the air inlet pressure $P$ to study the variance of the inner and outer diameters of the hollow-core double emulsions. We also apply the theoretical analysis^28^ that uses the following scaled variables: the product of the viscosity and the flowrate ratios of the two disperse phases, ${k}_{1}={\mu }_{2}{q}_{2}/{\mu }_{1}{q}_{1}$, and of the continuous and outer disperse phases, ${k}_{2}={\mu }_{3}{q}_{3}/{\mu }_{2}{q}_{2}$. The flow rate of air, ${q}_{1}$, is estimated by measuring the volume of the hollow-core droplets in the reservoir and the production rate at the step. We note that this indirect method for estimating ${q}_{1}$ can only be readily applied in the SE regime, where the droplets are highly monodisperse. The relation between the inlet air pressure and its flow rate is not trivial due to the relatively high (pressure-dependent) solubility of air in the PDMS material, resulting in some air escape. Detailed analysis is provided in the Supplementary document and supported by Supplementary Fig. S5, which estimates air leakage. However, this air deficit does not affect our analysis of the droplet size below, where the air flow rate is calculated *a posteriori* upon measuring the generation frequency and the core size of the double emulsion droplets.

**Fig. 3:**
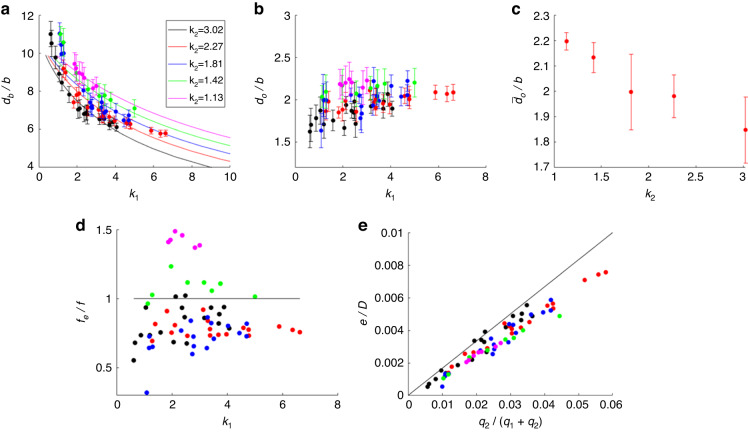
Droplet size upon varying flow rates. **a** Normalized bubble (air core) diameter ${d}_{b}/b$ versus ${k}_{1}$ for fixed ${k}_{2}$. Dots are experimental data, and the solid lines are the theoretical predictions of Eq. 1 with a multiplicative correction Factor ≈4. **b** Normalized oil (FC-40) droplet diameter ${d}_{o}/b$ vs. ${k}_{1}$ at different fixed ${k}_{2}$ values. **c** Normalized oil (FC-40) droplet diameter averaged over the investigated air pressure range ${\bar{d}}_{o}/b$ vs. ${k}_{2}$. **d** The ratio of the experimental production rate of hollow core double emulsion droplets, ${f}_{e}$, with the estimated rate of production of the resulting oil droplets, $f={q}_{2}/{V}_{o}$, vs. ${k}_{1}$, at fixed ${k}_{2}$ values, the solid line stands for the constant value of 1. **e** Normalized shell thickness by the outer diameter of the hollow-core double emulsion, $e/D$, plotted vs. the ratio ${q}_{2}/({q}_{1}+{q}_{2})$, the solid line is the linear line passing through the origin with a slope 1/6. Colors in (**a**), (**b**), (**d**) and (**e**) correspond to different ${k}_{2}$ values: ${k}_{2}=3.02$ (black); ${k}_{2}=2.27$ (red)$;$ ${k}_{2}=1.81$ (blue); ${k}_{2}=1.42$ (green); ${k}_{2}=1.13$ (magenta)

As shown in Fig. 3a, at a fixed oil flow rate, ${q}_{2}$ (i.e., at a fixed ${k}_{2}$), the measured diameter of the bubbles (air cores of the double emulsions as shown in Fig. 1d) normalized by the Hele-Shaw channel height, ${d}_{b}/b$, decreases with increasing ${k}_{1}$. Here, the subscript “*b*” stands for bubble. Since the oil shell is ultrathin, the air core diameter ${d}_{b}$ cannot be distinguished from the outer diameter of the hollow core shell emulsion, *D*. However, *D* can be calculated based on the diameter of the oil (FC40) droplets, ${d}_{o}$, that we finally collect from the reservoir. Figure 3b shows that at fixed ${k}_{2}$, ${d}_{o}$ is a slightly increasing function of ${k}_{1}$, indicating that ${d}_{o}$ is weakly dependent on the inlet air pressure. The oil droplet diameter averaged over the investigated range of the air inlet pressure, ${\bar{d}}_{o}/b$, is plotted vs. ${k}_{2}$ in Fig. 3c, suggesting that, as anticipated, it is an increasing function of the oil flow rate ${q}_{2}$. The hollow-core double emulsions form a closely packed array (Fig. 1d), which makes it difficult to accurately probe the generation rate of the resulting oil droplets. However, the generation rate can be readily estimated as $f={q}_{2}/{V}_{o}$, where ${V}_{o}$ is the volume of a single oil droplet. Then, *f* can be compared with the experimentally measured rate ${f}_{e}$ of the hollow-core double emulsions at the step. The frequency ratio ${f}_{e}/f$ at fixed ${k}_{2}$ values is plotted vs. ${k}_{1}$ in Fig. 3d, demonstrating a rather narrow distribution centered at approximately 1. This observation suggests that on average, a single hollow-core double emulsion droplet yields one oil droplet following dissolution of the air core. Deviations from unity, i.e., ${f}_{e}\, < \,f$, for larger values of ${k}_{2}$ (i.e., smaller ${q}_{2}$), suggest that more than one oil droplet is obtained from one hollow-core double emulsion on average, probably due to oil film rupture during step emulsification. On the other hand, the data showing ${f}_{e}\, > \,f$ (for a smaller value of ${k}_{2}$, i.e., larger ${q}_{2}$), indicates that more than one hollow-core double emulsion droplet contributes to a single oil droplet, probably due to coalescence events. However, the distribution of ${f}_{e}/f$ approximately 1 is quite narrow, suggesting that both $f$ and ${f}_{e}$ provide quite accurate estimates of the production rate of the oil droplets. Applying this argument together with the mass conservation, i.e., the shell volume ${V}_{{shell}}=\pi {d}_{b}^{2}e$, where *e* is the shell thickness, being equal to the resulting oil droplet volume ${{V}_{{shell}}\approx V}_{o}=\pi {d}_{o}^{3}/6$, one can compute the outer diameter *D* of the hollow-core double emulsion. We next compare ${d}_{b}/b$ (Fig. 3a) with the theory developed for the minimal diameter of the double emulsion droplet generated with the triphasic liquid step-emulsification^28^:

1 $$\frac{D}{b}=\frac{3\left({u}^{2}+\gamma \right)}{\left(1+\frac{1+{k}_{{1}_{{eff}}}}{\beta }\right){u}^{3}+\left(1+\frac{1+{k}_{{2}_{{eff}}}}{\beta }\right)\gamma }$$

where $u={\left(\frac{{q}_{1}}{{q}_{1}+{q}_{2}}\right)}^{1/3}={\left(\frac{1}{1+\lambda {k}_{1}}\right)}^{1/3}$, with $\lambda =\frac{{\mu }_{1}}{{\mu }_{2}}$, ${k}_{{1}_{{eff}}}={k}_{1}(1+{k}_{2})$, and ${k}_{{2}_{{eff}}}=\frac{{k}_{1}{k}_{2}}{1+{k}_{1}}$. The theory also predicts the scaled bubble diameter, ${d}_{b}/b={uD}/b$. Our experimental data agree with the theory with a multiplicative correction factor of ≈4, indicating that the step-emulsification with gas as the inner disperse phase for generating bubbles engulfed by a liquid shell follows the same scaling as for liquid‒liquid double emulsions. It can also be shown that in the SE regime, the ratio between the volumes of the oil shell and the hollow-core double emulsion droplet, $\frac{{V}_{o}}{{V}_{D}}$ is linearly proportional to $\frac{{q}_{2}}{{q}_{1}+{q}_{2}}$. Therefore, the shell thickness normalized by the outer diameter scaling is $\frac{e}{D} \sim \frac{{q}_{2}}{6\left({q}_{1}+{q}_{2}\right)}$ ^28^. As shown in Fig. 3e, the experimental data for $e/D$ plotted vs. ${q}_{2}/({q}_{1}+{q}_{2})$ fall on a straight line with a slope of $\approx 1/6$. The slight deviation from the 1/6 slope may be attributed to the formation of a satellite drop^13^ of oil at the step during the SE, whose volume is not accounted for in the shell volume. Some satellite drops can be observed in Supplementary Fig. S7b, while their contribution to the net volume is negligible.

#### Comparison with alternative microfluidic methods

**Fig. 4:**
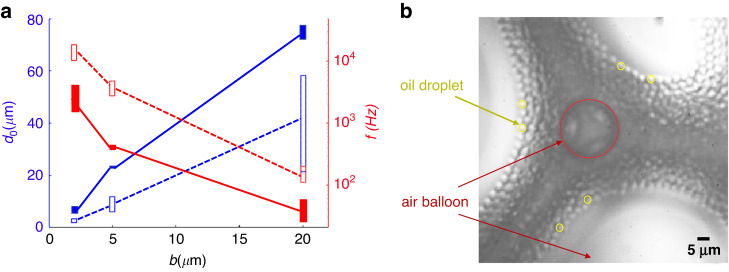
Oil droplet size and production rate by the air-assisted SE method in comparison with the biphasic coflow SE method. **a** Oil (FC40) droplet diameter ${d}_{o}$ at three different channel heights, *b*, and the corresponding droplet production rate, *f*. The height of the rectangle represents the range of the oil droplet diameter (blue) and of the frequency (red) upon varying the inlet air pressure and oil flow rate. Filled rectangle for the biphasic coflow SE method and empty rectangle for the air-assisted SE method. The solid lines and the dashed lines are drawn for eye guidance. **b** The micron-size oil (FC-40) droplets downstream in the reservoir, along with large air “balloon” bubbles supposedly following Ostwald ripening

It is well established that in biphasic coflow step-emulsification, the droplet diameter is 3.5–7 times the Hele-Shaw channel height, and the production rate increases upon downscaling the channel height^26^. In this work, we use devices with three different channel heights (b = 1.8 μm, 5 μm, and 20 μm) to generate hollow-core double emulsions at the step and subsequently form smaller single oil droplets due to air dissolution. We measure the droplet production rate and size at various flow rates of the outer disperse oil ${q}_{2}$ and the inlet air pressure *P*. The absolute inlet air pressure *P* varies with the channel height *b*, which is 100–160 mbar for channels with *b* = 20 μm, 550–800 mbar for *b* = 5 μm, and 1100–1600 mbar for *b* = 1.8 μm. It is also shown that for both biphasic (air/water) SE and triphasic (air/oil/water) SE, there is only marginal dependence of droplet size on ${q}_{2}$ and *P* (see Supplementary Fig. S6). Therefore, we take all the diameters and rates of droplets made in the investigated range of ${q}_{2}$ and *P* and plot the interval values in Fig. 4a. We find that air-assisted SE has throughput an order of magnitude higher than biphasic coflow SE at a fixed flow rate of oil ${q}_{2}$. In addition, the droplet size resulting from air-assisted SE overcomes the limitation of biphasic coflow SE (i.e., $d \sim 3.5b-7b$), and it reaches as low as $d \sim 1.7b.$ A detailed comparison between air-assisted SE and previous works dedicated to the generation of micron droplets is shown in Table 1. First, air-assisted SE offers the best throughput per single channel but falls behind methods with many parallel channels^43–46^. However, air-assisted SE has the potential to be parallelized, similar to the parallelization of flow-focusing and T-junction devices, demonstrating the generation of droplets in the THz range^44^. Second, the smallest droplet diameter measured in this work is ~2.7 $\upmu {\rm{m}}$ at a production rate of $1.88{\times 10}^{4}$ drops/sec using a channel with height *h* = 1.8 μm. A comparison with the previously reported biphasic coflow SE method, which generated droplets with similar diameters and production rates of $5\times {10}^{3}$ drops/sec and used a device with a height of 1 μm^26^, unequivocally shows that the air-assisted SE method is advantageous in terms of throughput and geometry, as it does not require ultrashallow (submicron) channels to generate micron-sized droplets. Submicron channels possess high hydraulic resistance (inversely proportional to ${b}^{3}$), requiring high operating pressures. In addition, fabrication of submicron channels is a challenging undertaking. Submicron-depth microchannels fabricated from PDMS using conventional soft lithography are prone to collapse due to the pressure buildup required to drive fluids, while rigid silicon or glass microchannels require sophisticated microfabrication procedures. In the air-assisted SE method, integrating an extra inlet channel for the air into the device (as compared to conventional biphasic SE) is only of an incremental complexity, whereas submicron channel depth (for conventional biphasic SE) and submicron orifice (for flow focusing) present a substantial technical challenge. Our estimates suggest that reducing the channel height down to 500 nm in air-assisted SE would yield the generation of submicron-sized droplets at even higher throughput. One potential drawback of the air-assisted SE is that the corresponding deviation (CV) is higher than in the alternative methods, as shown in Table 1. This may be caused by the fluctuation of the supplying air flow, which may be avoided by controlling the air flow rate rather than pressure. The stability of the triphasic air-assisted coflow through the Hele-Shaw channel is comparable to that of the conventional all-liquid biphasic SE method. In summary, gas-assisted SE provides an efficient concept to produce droplets at the micron scale with competitive throughput per single channel, while it does not rely on sophisticated nanofabrication and high operating pressure. More importantly, since air (or, e.g., nitrogen) is inert with respect to many liquids, the method is less selective in terms of the chemical composition of the liquid phases compared with other methods based on solvent extraction.

**Table 1:** Comparison of droplet size and production rate in this work vs. previous microfluidic techniques designed for high throughput production of micron- and submicron-sized droplets

Method	${d}_{o}$ (CV) [μm] (%)	Rate *f* [drops/sec] (single channel)	Rate *f* [drops/sec] (parallelized channel)	Channel depth *b* [μm]	Microfabrication method
Co-flow SE^27^	0.4	~10^4^	/	0.1	Glass etching
Co-flow SE^26^	0.9 (1%)	1.5 × 10^4^	/	0.42	PDMS
Cross-flow submicron channel^48^	1.4 (16.7%)	15	~10^4^	0.32	Silicon
Air-assisted SE (this work)	2.7 (11%)	1.88 × 10^4^	/	1.8	PDMS
Straight-through Microchannel^49^	4.4 (<6%)	>30	~3 × 10^4^	1.7	Silicon
Microchannels^43^	5	134.5	2 × 10^6^–2 × 10^7^	1.8	Silicon
SE in array^50^	8	270	6.7 × 10^4^	2	PDMS
Multi-EDGE^46^	10 (<10%)	>1800	4.8 × 10^6^	2	Silicon
Microchannels^45^	14.1 (<3%)	2.8	~5 × 10^3^	4	Silicon
Flow focusing and T-junction in array^44^	24.5 (2.4%)	~10^4^	~10^8^	22.5	Silicon + glass

### Air-assisted emulsification of highly viscous liquids

**Fig. 5:**
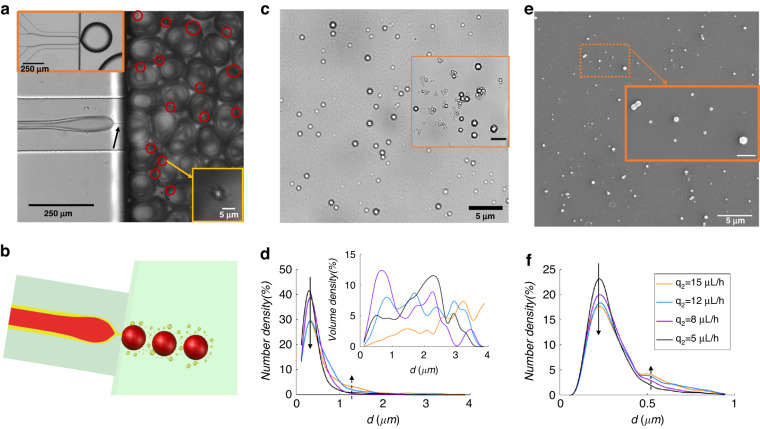
Formation of ETPTA droplets at the micron and submicron scales by gas-assisted step-emulsification. **a** Experimental image of air-ETPTA compound thread pinching synchronously at the step. The high viscosity of ETPTA (64 mPa·s) leads to filament formation, as indicated by the black arrow. Some visible ETPTA microdroplets are marked by red circles, and the magnification of one microdroplet is shown in the lower-right inset. The upper-left inset shows ETPTA “balloon” droplet formation without gas assistance at the same flow rate of ETPTA (${{{q}}}_{2}$); no small droplets of ETPTA can be generated in this regime. **b** Sketch of the flow regime and droplet generation at the step. **c** UV-cured ETPTA beads taken from the supernatant after being size sorted by sedimentation. Inset: a polydisperse distribution of the ETPTA beads before sedimentation, scale bars stand for 5 μm. **d** Number density of the size of the UV-cured ETPTA beads before sedimentation. Inset: the volume density of the beads before sedimentation. **e** SEM image of the UV-cured ETPTA beads taken from the supernatant after sedimentation; the scale bar in the inset represents 1 μm. **f** Number distribution of the size of the colloidal beads after sedimentation

Fluids with high viscosity are usually difficult to emulsify into droplets due to slow pinching. As shown in the upper-left panel of Fig. 5a, ETPTA (Newtonian fluid with viscosity 64 mPa·s) is injected as the disperse phase at a flow rate of ${{12}}\,{{\upmu }}{\rm{L}}/{\rm{h}}$, and SDS aqueous solution at 0.2% (w/w) is injected as the continuous phase. Large “balloon” drops are formed, and the SE regime generating small droplets cannot be observed. This is caused by the high viscosity of the disperse phase, leading to capillary number ${{Ca}}$ higher than the critical value at the transition between SE and BE regimes^19^. To remain in the SE regime, one can decrease the flow rate of the ETPTA; however, this would reduce the throughput. Air-assisted SE can be used to circumvent these obstacles, with air and ETPTA coinjected as the inner and outer disperse phases, respectively, as shown in Fig. 5a and sketched in Fig. 5b. The compound air-ETPTA thread pinches at the step generating air bubbles and ETPTA micron-sized droplets (see the red circles and the magnified image in the inset of Fig. 5a), while submicron-sized droplets are not visible in Fig. 5a. The critical (i.e., minimal) inlet air pressure, above which pinching of the air-ETPTA compound thread occurs at the step, increases with the ETPTA flow rate, ${q}_{2}$. As shown in Supplementary Fig. S8, the critical air pressure is also an increasing function of the viscosity of the liquid disperse phase.

High-speed imaging reveals the formation of filaments connecting the already formed ETPTA droplets and the ETPTA thread upstream of the step, as indicated by the black arrow in Fig. 5a. This filament formation is due to high-viscosity fluid under a high elongation rate, and it will then retract and evolve into ETPTA droplets, reminiscent of the satellite drops during filament breakage. The filament eventually pinches and breaks into several smaller droplets of the ETPTA.

The fluids at the exit of the channel are collected into a reservoir, which includes the bubbles and the ETPTA drops. Since the surface tension between the ETPTA and the air is ${\gamma }_{12}={37.02}$ mN/m, that of ETPTA/SDS solution is ${\gamma }_{23}={4.25}\,{\text{mN}}/{\text{m}}$, and SDS solution/air ${\gamma }_{13}={35.41}$ mN/m, the spreading parameter ${S}_{3}\, < \,{0},\,{S}_{1}\, <\, {0}\,{\text{and}}\,{S}_{2}\, < \,{0}$, so that no hollow-core double emulsion is formed at the step as we observed, the ETPTA droplets and the air bubble form Janus structures, or individual ETPTA drops and air bubbles. The air bubbles disappear within 2 h by diffusion under ambient atmosphere or by bubble bursting, leaving the ETPTA droplets suspended in SDS solution. After ultraviolet curing, the ETPTA beads are subjected to dynamic light scattering (DLS) measurements; however, DI water, SDS aqueous solution and the ETPTA nanosuspension all show a peak on the order of several hundred nanometers, so we cannot distinguish the presence of ETPTA nanoparticles by DLS. Alternatively, we measured the ETPTA beads under an optical microscope (Fig. 5c inset), showing a visually polydisperse distribution of bead sizes at the micron and submicron scales. Using x1500 magnification, we recognized beads at the 100 nm scale. More than 3000 particles are measured from pictures taken at 10 different positions under a microscope, and the droplet size distribution in number density is plotted in Fig. 5d. A large peak is situated at approximately 325 nm diameter, and there is a negligible number of beads at the micron scale. The diameter at the peak number density is not significantly affected by the flow rate of ETPTA; however, the peak number density of submicron beads decreases with increasing ${q}_{2}$, as shown by the black solid arrow in Fig. 5d. Moreover, a higher flow rate of ETPTA leads to a larger number density of beads at the micron scale, as shown by the black dashed arrow. The inset of Fig. 5d shows the distribution in volume for the size of the droplets, and an increase in the ETPTA flowrate tends to contribute most of the ETPTA fluid volume in forming droplets at the micron scale, as shown by the orange curve with ${{{q}}}_{2}={15}\,{{\mu }}{{L}}/{{h}}$ (see Fig. 5d inset). However, decreasing the flowrate of the ETPTA leads to the contribution of more ETPTA volume in forming submicron droplets (purple curve in Fig. 5d inset). Figure 5d and the inset suggest that a smaller flow rate of ETPTA is advantageous for generating submicron droplets.

The collected mixture (i.e., the bubbles and ETPTA beads) was left for sedimentation for 2 days, and most micron-sized beads sediment due to gravity (density of ETPTA${{\rho }}={1.1}\,{{g}}/{{{cm}}}^{3}$), whereas the thermal agitation of the submicron beads was strong enough to overcome gravity. We observed the supernatant under a microscope (Fig. 5c), and colloidal beads were observed to undergo Brownian motion. The same sample was subjected to SEM observation (Fig. 5e). More than 5000 particles from pictures taken at 20 different positions under a microscope were measured for statistical analysis, and the number density of the bead size in the supernatant part is shown in Fig. 5f, which shows a large peak in the vicinity of 230 nm. An increase in the flowrate of ETPTA (${{{q}}}_{{{2}}}$) leads to a decreasing number density of the 230 nm beads, as shown by the solid arrow. However, we can obtain a greater number of relatively large colloidal droplets by imposing a higher ETPTA flowrate, as shown by the dashed arrow. To confirm that the inclusions observed via microscope and SEM are actually ETPTA beads and not impurities, we dried both DI water and SDS solution on a glass slide under the optical microscope, both of which showed no beads. Therefore, we confirm that the beads observed in the ETPTA experiments are formed from ETPTA.

The air-ETPTA compound thread is subject to fast oscillations during step-emulsification, resulting in strong elongational stretching of the ETPTA filament beyond the step (see the black arrow in Fig. 5a). The high viscosity increases the internal flow resistance so that the liquid filament thins continuously until capillary fragmentation takes place^13^. In this experiment, the colloidal droplets are likely to be satellite or subsatellite droplets during filament pinching^12^, and the droplet size distribution may depend on the viscosity ratio between the disperse and continuous phases. Colloidal droplets may also be produced by bubble bursting, and during the process, the retraction of the aqueous film provides a strong shear effect on the oil droplets to produce colloidal droplets^47^. Therefore, with the assistance of air injection as the inner disperse phase, we achieved the formation of colloidal droplets of high-viscosity fluid in the step-emulsification device. The gas-assisted SE method generates more uniform colloidal droplets of high-viscosity fluid in comparison to the flow focusing method, in which large primary drops are first formed, providing conditions for thread stretching, which allows tiny droplet formation^9^. The tip-streaming method is restricted to low viscosity and low flowrate of the disperse phase^12^, while the gas-assisted step-emulsification is not (or less) affected by this limitation.

## Conclusion

The high-throughput production of highly monodisperse micron- and submicron-sized droplets is required in many biomedical applications, such as ultrasound imaging and drug delivery. Conventional coflow step-emulsification methods^26–28^ have been demonstrated to generate highly monodisperse emulsions at high production rates; however, the generation of micron-sized droplets would require submicron-height microchannels, which must be fabricated from a rigid material (e.g., glass or silicone) due to high hydraulic resistance, rendering microfabrication complex and expensive in comparison to PDMS-based soft lithography. Conventional microfluidic settings (such as flow-focusing) can theoretically generate micron-sized droplets by reducing the channel dimension, but they are subjected to the same technical difficulties described above.

In coflow step-emulsification, generation of small droplets requires the capillary number not to exceed some critical value, beyond which only large “balloon” droplets (of the size much larger than the channel height) can be generated. Since the capillary number is proportional to the flow rate and viscosity, it limits the single channel throughout, particularly for high-viscosity fluids. Some biological and chemical fluids do have high viscosity and cannot be easily emulsified.

We propose an alternative method of gas-assisted coflow step-emulsification, which is able to generate gas-in-oil-in-water hollow-core double emulsions, which act as precursors for micron-sized oil-in-water emulsions. Following Ostwald ripening and dissolution of the air cores, monodisperse micron-sized oil droplets are formed, with a minimal diameter reaching ~1.7 times the channel height, twice as small as in the standard biphasic step-emulsification. Due to the low viscosity of the air, the production rate is increased by an order of magnitude in comparison with the standard step-emulsification method. At the same time, this method can be performed using microchannels ($b\,\gtrsim \,1\,\upmu {\rm{m}}$), bypassing the need for complex nanofabrication of submicron channels and high operating pressures. In particular, we demonstrate that highly viscous fluids, such as ETPTA fluid with a viscosity of 64 mPa·s, can be effectively emulsified using the proposed method of air-assisted SE, which benefits from a considerable reduction in the effective capillary number due to the low viscosity of air. The submicron droplet size (controlled by the oil flow rate) is distributed at approximately 230 nm. The use of inertial gas (e.g., air) renders the proposed method attractive for high-throughput generation of micron- and submicron-sized droplets from a wide variety of biological fluids of different chemical compositions and viscosities.

## Materials and methods

### Materials

The SDS aqueous solution was prepared from sodium dodecyl sulfate (SDS, from Meryer) dissolved in deionized water (from Greagent) at two different concentrations. A 2.5% (w/w) SDS aqueous solution was used as the continuous phase in the experiment of generating hollow-core double emulsions with Fluorinert FC40 oil (purchased from 3 M). A 0.2% (w/w) SDS aqueous solution was used as the continuous phase in the experiments of generating the trimethylolpropane ethoxylate triacrylate (ETPTA) droplets. ETPTA (from Aladdin) was mixed with 0.5% (w/w) of photoinitiator 2-hydroxy-2-methyl-1-phenyl-1-propanone (from Sigma‒Aldrich). The surface tension between the liquids was measured using the pendent drop method with an optical contact angle meter Dataphysics OCA20.

### Microfluidic device

Microfluidic step-emulsification devices were fabricated with the standard soft lithography method. The silicon wafer was prepared with SU-8 photoresist under UV exposure and possessed two different heights realized by a mask aligner in the lithography machine. The microchip was prepared from polydimethylsiloxane (PDMS, Dow Chemical), and a mixture of PDMS and the curing agent at a 10:1 ratio was poured onto the silicon wafer and then heated in an oven at 80 °C for 2 h. The PDMS block was sealed with a glass slide with plasma (Harrick PDC-32G-2) for 35 s, which rendered the channel walls hydrophilic. The experiments were conducted immediately after the plasma treatment, which preserved the hydrophilicity of the PDMS channel walls. A stable generation of droplets for 1 h and 20 min is demonstrated in Supplementary Movie S2, while our experience suggests that hydrophilicity lasts even longer. The inlets were made using hole punchers, and the outlet was made by knife cutting for a larger contact area with the ambient atmosphere.

The device has three inlets for the three fluid phases, two junctions, and a shallow (Hele-Shaw) microchannel, which is connected with a wide and deep reservoir (Fig. 1a). We used devices with three different geometries of the Hele-Shaw channel: width $w=200\,\upmu {\rm{m}}$, height $h=20\,\upmu {\rm{m}}$; $w=150\,\upmu {\rm{m}}$, $h=5\,\upmu {\rm{m}}$; and $w=20\,\upmu {\rm{m}}$, $h=1.8\,\upmu {\rm{m}}$. Although the Hele-Shaw channels had a high aspect ratio $\beta =w/b\, > \,10$, collapse did not occur unless the channel height was $\lesssim 1\,\upmu {\rm{m}}$. In addition, the collapse can possibly be circumvented by hardening the PDMS material, elongating the baking time, or increasing the baking temperature to 90 °C.

### Fluid flow and recording technique

The liquids were injected by syringe pumps (Longerpump), and the air was injected by a pressure pump (Fluigent). We first injected air to prevent liquids from entering the air inlet channel and then the SDS aqueous solution to keep the hydrophilicity of the channel intact and the oil. The flow was observed under an inverted transmission microscope (Nikon Ti2-U) and filmed by a high-speed camera (Photron Fastcam mini).

### Droplet generation analysis

The liquids were injected by controlling the flowrates of the oil and aqueous phase, whereas the injected air was controlled by pressure. The flow rate (required for comparison to theoretical predictions) was calculated based on measuring the size of the hollow-core double emulsion and production frequency measured in the experiments. The droplet sizes of the hollow-core double emulsions and of oil droplets were measured by ImageJ software. In the case of ETPTA droplet formation, we collected the formed mixture of bubbles and droplets at the exit of the channel and exposed it to UV for 20 min for solidification. Then, we left it for 2 days for the beads to sediment due to gravity. We measured the supernatant by dynamic light scattering (DLS) and microscopy. For microscopic observation, we placed 20 μL of the supernatant onto a glass slide and observed the particles at ×1500 magnification. The water evaporated quickly, leaving the ETPTA beads fixed to the glass. In addition, we observed the ETPTA beads by scanning electron microscopy (SEM).

## Supplementary information

Supplementary Document

Supplementary Movie S1

Supplementary Movie S2

Supplementary Movie S3

Supplementary Movie S4
