# Supplementary material for: Gas-assisted microfluidic step-emulsification for generating micron- and submicron-sized droplets
Source: Microsyst Nanoeng. 2023 Jul 10;9:86. doi: 10.1038/s41378-023-00558-4 (PMC10330193; doi:10.1038/s41378-023-00558-4)
Supplement: Supplementary file 1 — Supplementary Document [file 41378_2023_558_MOESM1_ESM.docx]

Supplementary document

Gas-assisted microfluidic step-emulsification for generating micron- and submicron-sized droplets

Biao Huang, Xinjin Ge, Boris Y. Rubinstein, Xianchun Chen, Lu Wang, Huiying Xie, Alexander M. Leshansky* and Zhenzhen Li*

1. **Hele-Shaw hydrodynamics of the triphasic flow**

We use air as the inner disperse phase, FC40 oil as the outer disperse phase and SDS aqueous solution as the continuous phase. The three phases co-flow in a Hele-Shaw channel of width $w$ and height $b$, such that $w/b>10$. The channel walls are hydrophilic, so that they are well wetted by the continuous phase and poorly wetted by the outer disperse phase (oil). The cross-section of the confined compound thread in the Hele-Shaw channel is shown schematically in Figure S1a. The device design including the Hele-Shaw microchannel, the collecting reservoir, and the inlet channels is shown schematically in Figure S1b.


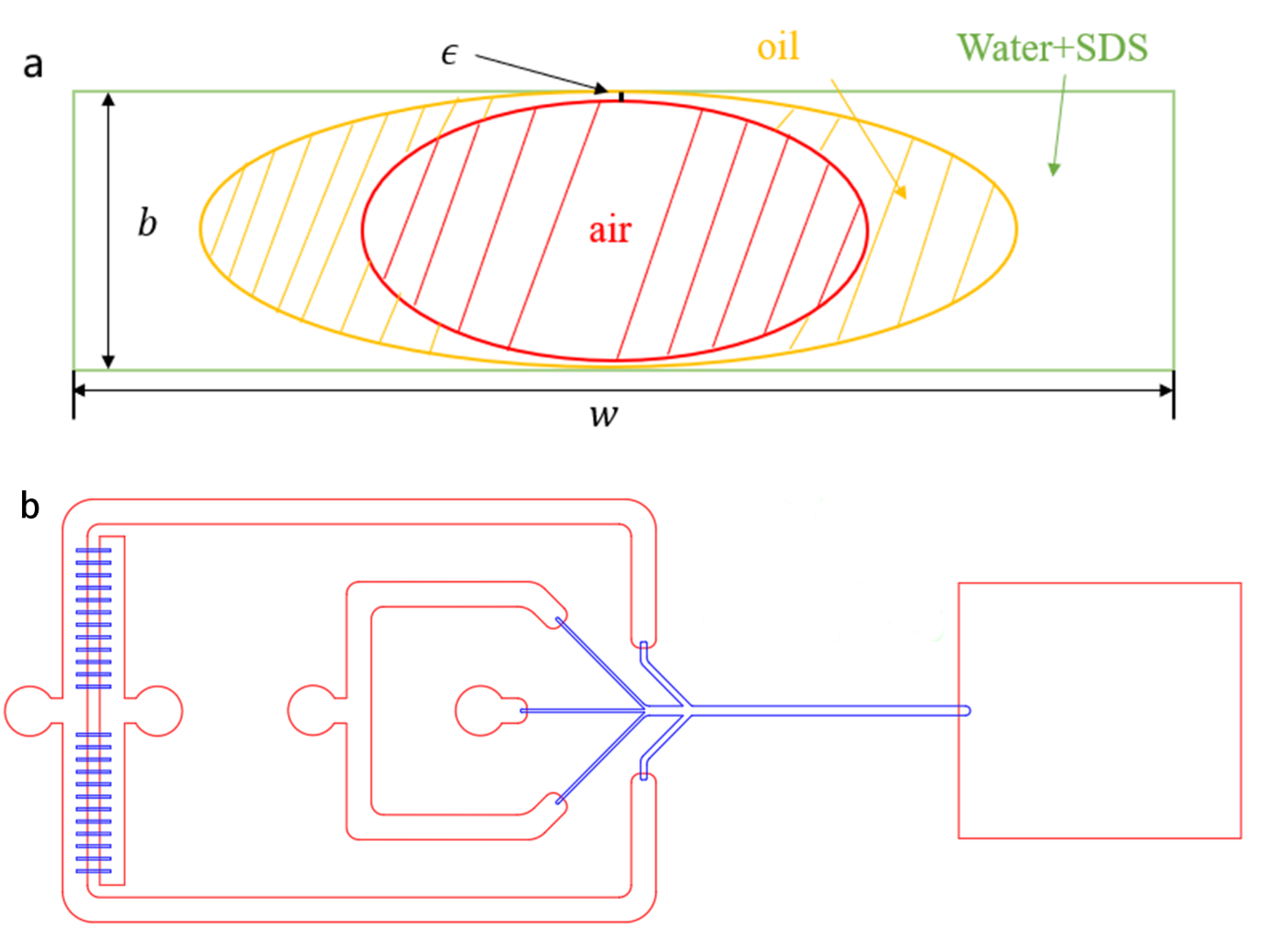


**Figure S1**: a) Cross-section of the Hele-Shaw channel with the confined compound air-oil thread; b) The schematic design of the microfluidic device of the double-layer micro-channel. Shallow Hele-Shaw channels (blue in the middle) have high aspect ratio $w/b>10$; filtering channels (blue on the left) have the same depth with the Hele-Shaw channels, and are aimed at filtering out the impurities suspended in the continuous phase; the inlet channels (red) have larger depth.

1. **Three regimes of single emulsion formation of FC40**

When the pressure of the air is too low to reach the first junction (J1), only the outer disperse oil and the continuous phase co-flow in the Hele-Shaw channel. Upon increasing the flow rate of oil ($q_{2}$), the biphasic T-junction emulsification (b-TE) in which the round oil jet breaks at the J2 by Rayleigh-Plateau instability turns into the biphasic step-emulsification (b-SE) whereas the confined oil thread breaks at the step at high frequency, followed by the biphasic balloon-emulsification (b-BE) whereas large (“balloon”) droplets are generated at the step, as depicted


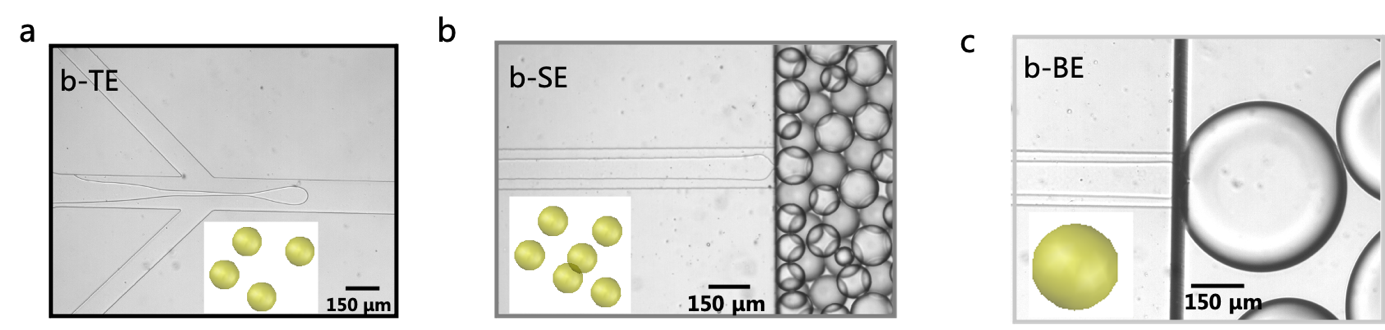
in Figures S2a-c, respectively.

**Figure S2:** Three dynamic regimes of the single emulsion generation, shown in the phase diagram ‎of Figure 2a in ‎the manuscript as black dots for a) biphasic T-junction emulsification (b-TE); (b) ‎dark grey dots for biphasic ‎step-emulsification (b-SE); (c) and light grey dots for biphasic balloon-emulsification (b-BE).

1. **Step-emulsification using different oils as the outer disperse phase**

Besides the FC40 oil, phenylmethyl silicon oil and mineral oil are both tested, showing that hollow-core microdroplets with uniform size distribution are generated. These droplets are formed in the step-emulsification (SE) regime followed by the air core shrinkage. A collection of air balloons and oil droplets are observed at the exit of the channel. The generated oil droplets and those with shrinking air cores are shown in Figure S3.


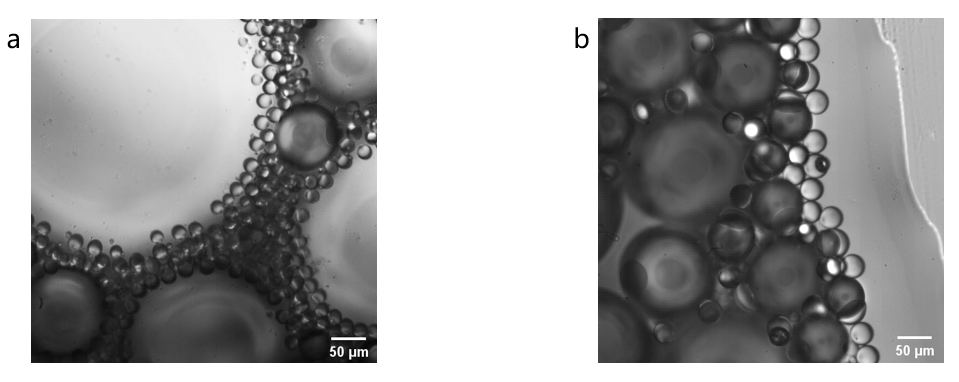


**Figure S3:** a): Hollow-core microdroplets filmed downstream in the reservoir in step-emulsification regime followed by the air core shrinkage: a) Phenylmethyl silicon oil is used as the outer disperse phase; the flowrates are: $q_{2}$=10 $\mu L/h$, $q_{3}$=600 $\mu L/h$ and the air inlet pressure $P$=104.1 $\mathrm{mbar}$; b) Mineral oil is used as the outer disperse phase; the flowrates are: $q_{2}$=20 $\mu L/h$, $q_{3}$=300 $\mu L/h$, and the air inlet pressure $P$=73.8 mbar. In both experiments, the Hele-Shaw channel has the width $w=200$ $\mu m$, and the height$b=20 \mu m$.

In order to demonstrate the broad applicability of the air-assisted method, we performed another experiment replacing FC40 oil with phenylmethyl silicon oil as the outer disperse phase. The resulting regime map in Figure S4a has a similar structure with the phase diagram using FC40 oil (see Figure 2a in the main text). However, there are some minor differences. The T-junction TE1 regime does not materialize with the silicon oil (see Figure 4Sa), instead, the breakup occurs at the 2nd junction (TE2). It means that the air-silicon oil compound thread breaks at the second junction (J2) of the channel, instead of J1. However, the TE1 and TE2 are both T-junction modes, and have similar mechanisms, that when the thread width is close to the channel depth, Rayleigh-Plateau instability develops leading to breakup of the thread at the junction. Another minor difference between the two phase diagrams is that when the air pressure is sufficiently low so that no air enters from the inlet channel, silicon oil does not break at the junction and no single T-junction mode is observed. This is because of the high viscosity of silicon oil, which forms a continuous thread in the Hele-Shaw channel, even at the lowest flow rate that can be imposed by the syringe pump. When compared to the phase diagram in Figure 2a, the regions corresponding to various emulsification regimes are shifted along both parameter axes (${P/{P^{*},} Ca}_{2}$), since the position and/or slope of boundaries separating them depend on the surface tension ratio $\gamma={\gamma_{23}}/{\gamma_{12}}$ (see Ref. [3]), which is different in both cases. However, the qualitative similarity between the two phase diagrams demonstrates the versatility of the method. The physical properties of fluids used in the experiments (Figure 2 and Figure S4) are given in the Supplementary Table S1.


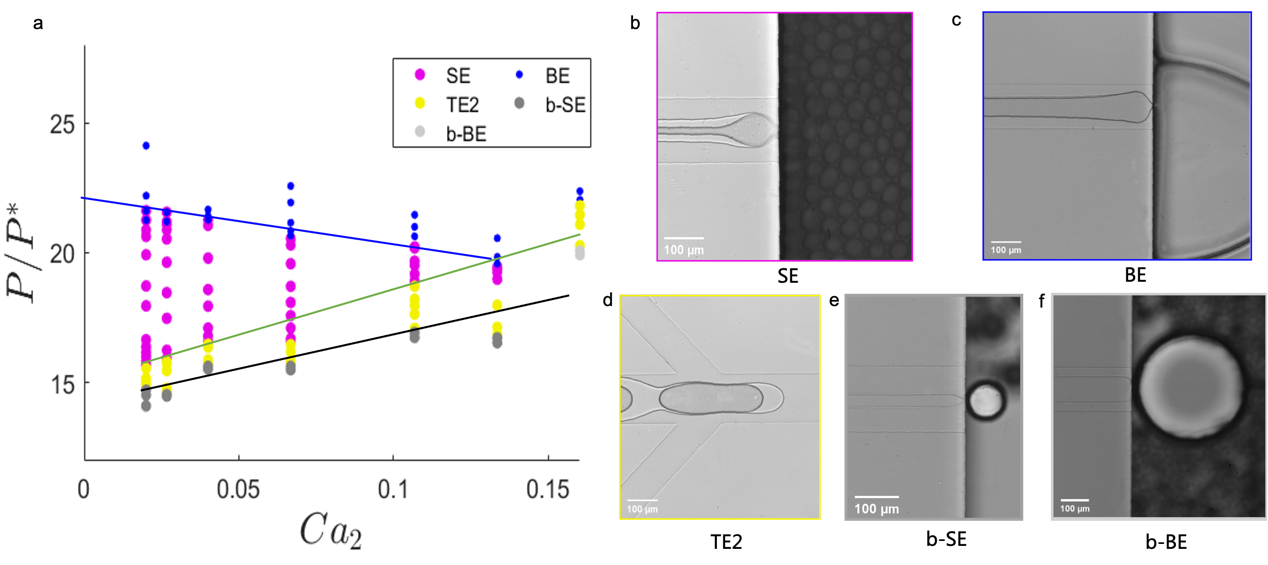


**Figure S4:** a) Phase diagram of the double hollow-core emulsification in the plane of the dimensionless air pressure $P/{P^{*}}$ and the oil capillary number, ${Ca}_{2}$. The Hele Shaw channel has the width $w=150 \mu m$, and the height $b=5 \mu m$. The aqueous (continuous) phase flow rate $q_{3}$=100 $\mu L/h$, the air as the inner disperse phase, phenylmethyl silicon oil as the outer disperse phase. Five emulsification regimes are observed: b) step emulsification (SE); c) balloon emulsification (BE); d) T-junction emulsification (TE2); e) biphasic step-emulsification (b-SE); f) biphasic balloon emulsification (b-BE).

**Table S1:** Physical properties of the fluids used in the experiments.

| Experiment | Phase | Fluid | Viscosity (mPa.s) | Surface tension (mN/m) |
| --- | --- | --- | --- | --- |
| Experiment 1  (Figure 2 in the main text) | inner disperse phase (1) | air | 0.0186 | $\gamma_{12}=16.09$ |
|  | outer disperse phase (2) | FC40 oil | 4.2 | $\gamma_{23}=15.62$ |
|  | continuous phase  (3) | SDS aqueous solution | 1 | $\gamma_{13}=33.63$ |
| Experiment 2  (Figure S4) | inner disperse phase (1) | air | 0.0186 | $\gamma_{12}=21.84$ |
|  | outer disperse phase (2) | phenylmethyl silicon oil | 31.4 | $\gamma_{23}=10.45$ |
|  | continuous phase  (3) | SDS aqueous solution | 1 | $\gamma_{13}=33.63$ |

1. **The relation between the inlet air pressure and its flow rate**

In this work, we control the inlet air pressure rather than its flow rate. There is nontrivial relationship between the applied pressure and the air flow rate in the channel, because PDMS is permeable to gas. The solubility of O_2_ and N_2_ in PDMS is ~0.18 cm^3^/(cm^3^$\cdot$atm) and ~0.09 cm^3^/(cm^3^$\cdot$atm) [1], respectively. Our operating gauge pressure is typically below 1 atm, so we assume the air solubility of $\sim0.2$ cm^3^(air)/cm^3^(PDMS). In a 1 cm thick PDMS block with volume ~10 cm^3^, the total capacity of soluble air is ~2 cm^3^. Given the typical flow rate of air of ${10}^{-9}$ m^3^/s and the typical time of emulsification $\sim$2 hours, the total injected volume of air would be $\sim2$ cm^3^, of the same order of magnitude as the capacity of PDMS block. So that the PDMS block acts as a large buffer pool for air injected to the Hele-Shaw channel. We conducted a well-controlled biphasic (air/water) co-flow experiment, in which we inject air as the disperse phase and SDS aqueous solution as the continuous phase. The air flow rate is controlled, instead of its inlet pressure. Figure S5 shows the dimensionless upstream width $\eta_{\infty}=l_{1}/w$ of air thread in the Hele-Shaw channel as function of the parameter $k=\mu_{2}q_{2}/\mu_{1}q_{1}$, with $\mu_{1}$ and $\mu_{2}$ being the viscosities of air and the SDS solution, respectively; $q_{1}$ and $q_{2}$ are the flow rates of the two phases. The flow rate of continuous phase $q_{2}$ varies as indicated in the legend of Figure S5. The flow rate of air $q_{1}$ ranges from 1 ml/h to 38 $ml/h$ for experiments with $q_{2}=100 \mu$l/h, and $q_{1}$ ranges from 1 ml/h to 6 $ml/h$ for the experiments with other $q_{2}$ values. The symbols in Figure S5 are the experimental results and the solid line stands for the theoretical prediction $\eta_{\infty}=1/(1+k)$ in Ref. 19. Apparently, the theory overestimates the width of the air thread in the Hele-Shaw channel, suggesting that the flow rate of the air through the Hele-Shaw channel is underestimated. The deviation between the experiment and the theory ranges between 1% to 37% depending on various parameters. A larger discrepancy is found at lower flow rate of the continuous phase, $q_{2}$ (lower values of $k$) probably due to wider air threads which has larger interfacial area facilitating air dissolution. Based on this well-controlled experiment, we can conclude that similar air loss occurs in the air-assisted triphasic setup. However, this air loss does not affect our quantitative analysis concerning the droplet size in Figure 3a, in which we control the inlet air pressure, as the flow rate of air in the Hele-Shaw channel is calculated *a* *posteriori* upon measuring the production frequency and the core size of the emulsion droplets.


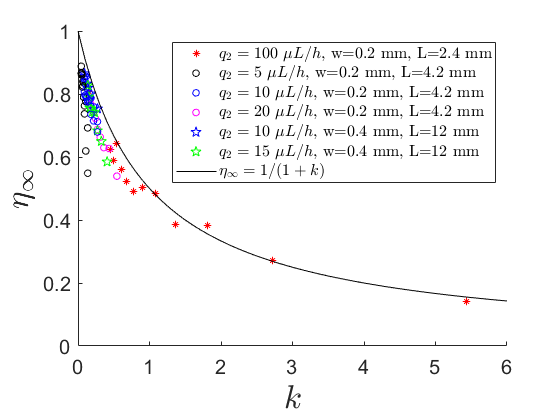


Figure S5: Biphasic co-flow experiment with air as a disperse phase and aqueous SDS solution as the continuous phase, the dimensionless upstream width $\eta_{\infty}$ of air thread in the Hele-Shaw channel vs. $k$. The symbols are the experimental results, and the solid line corresponds to the theory. In the legend, $q_{2}$ stands for the flow rate of continuous phase. The Hele-Shaw channels used in this experiment had the fixed depth of $15 \mu m$, two different widths $w$ and three different lengths $L$, as indicated in the legend.

**5.The throughput and droplet size in air-assisted step-emulsification**

In order to study the effect of the air-assisted step-emulsification on the throughput and oil droplet size, we tested droplet formation at various flow rate of outer disperse oil $q_{2}$, and the air pressure $P$, and we used 3 different channels with height: 1.8$\mu m$, 5 $\mu m$ and 20 $\mu m$. The geometric dimensions of 3 different channel design are listed in Table S2. The results are displayed in Figure S5. The $q_{2}$ and $P$ do not have significant influence on throughput and on final oil droplet size, for all the three devices. However, comparing the biphasic co-flow SE and air-assisted SE, the throughput can be increased with one order of magnitude by the air-assisted SE for a fixed value of $q_{2}$. In terms of final oil droplet size, the standard biphasic co-flow SE forms droplet with diameter 4-5 times the channel height, whereas the air-assisted SE produces droplets with the diameter about twice the channel height. Note that the droplets formed from air-assisted SE are less uniform than in the standard all-liquid biphasic SE method. The measured coefficient of variance CV was 11% for droplets generated by the device with channel height 1.8$\mu m$, CV=10% with the channel height of 5 $\mu m$ and CV=9% for channel height of 20 $\mu m$. The CV does not vary significantly with the droplet size.

We calculate the flow rate of oil phase $q_{2}$ through the drop size and frequency that we measure at the step and compare it with the flow rate prescribed by the syringe pump, the former is only 60-80% of the latter. This indicates that not all injected oil arrives to the oil droplets via the hollow-core double emulsions shown in Figure S6a. We collect the mixture of the drops and bubbles, leave the emulsion until all bubbles dissolve, and observe the emulsion under the microscope. We observe oil droplets with diameter about twice of the channel height$d\sim2b$ as well as some tiny oil droplets that are order-of-magnitude smaller, as shown by Figure S6b. The presence of tiny oil droplets may explain the above mismatch in the flow rate of oil, they are satellite droplets accompanying the hollow-core double emulsions formation when the air-oil compound thread pinches at the step.

**Table S2:** The geometric dimensions of 3 different microchannel designs in Figure S1b.

|  | Hele-Shaw channel (blue) | | Inlet channel (red) | |
| --- | --- | --- | --- | --- |
| Channel | Height ($\mu m$) | Width ($\mu m$) | Height ($\mu m$) | Width ($\mu m$) |
| 1 | 20 | 200 | 200 | 2000 |
| 2 | 5 | 150 | 200 | 400 |
| 3 | 1.8 | 20 | 40 | 200 |


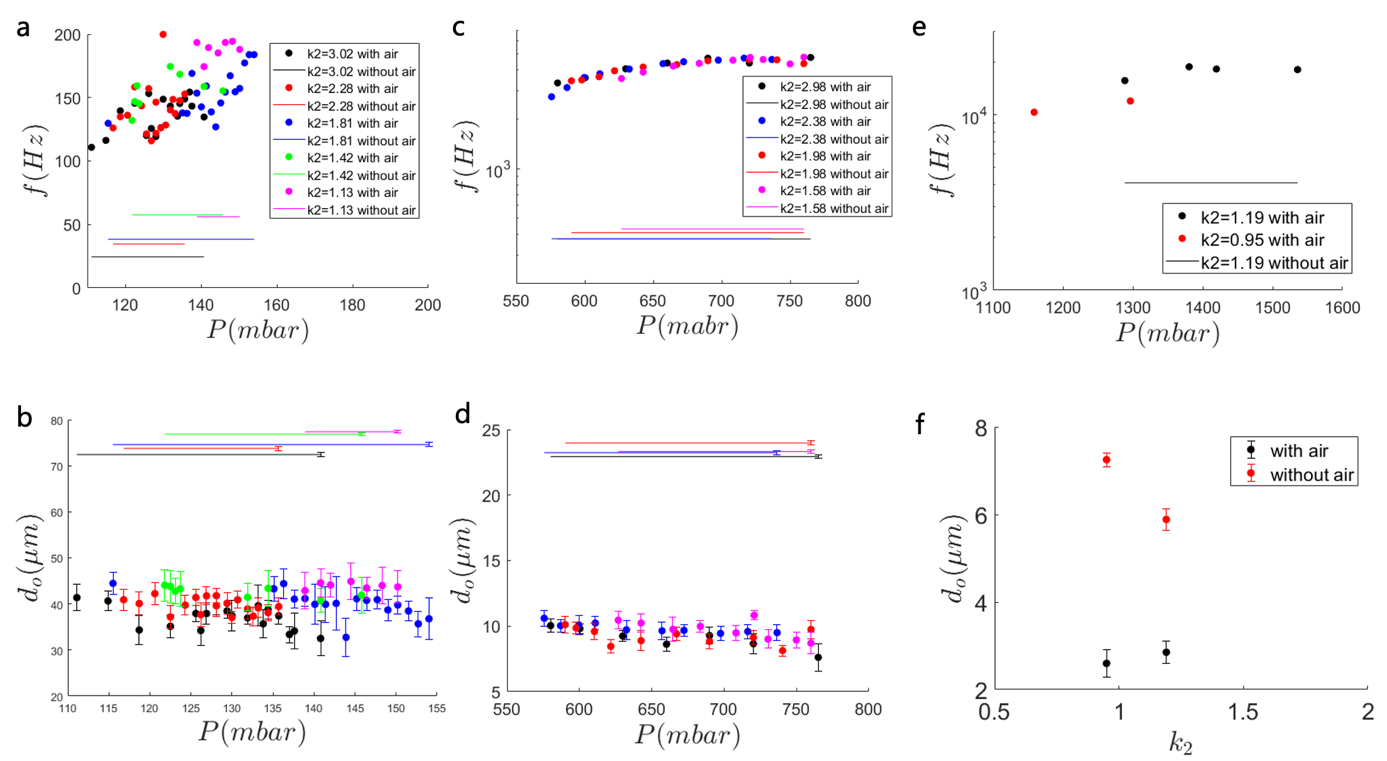


**Figure S6.** The frequency ($f$) and size ($d_{o}$) of FC40 droplets versus the air inlet pressure $P$ at fixed $k_{2}$ values. a) and b) for channel height at 20 $\mu m$, $q_{3}=200 \mu L/h$. c) and d) for the channel height at 5 $\mu m$, $q_{3}=100 \mu L/h$. e) and f) the channel height at 1.8 $\mu m$,$q_{3}=20 \mu L/h$. In a)-d), results using the method of air-assisted SE (dots) are compared with results using the standard biphasic co-flow SE method (lines) at the same $k_{2}$. Black dots in f) represent the average value of $d_{o}$ over an investigated range of air pressure at a fixed $k_{2}$.


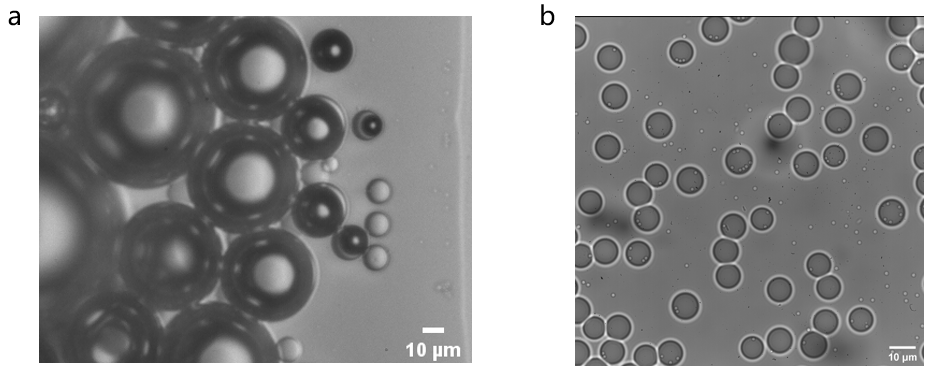
**Figure S7.** FC40 droplets formed in the channel with width 150 $\mu m$ and height 5$\mu m$. a) Hollow-core double emulsion droplets and single emulsion oil droplets (on the right) following dissolution of the air core; b) The collected oil single emulsions after complete dissolution of the air cores and tiny oil droplets with order-of-magnitude smaller diameters.

**6. Generating high viscosity droplet via air assisted step-emulsification**

In the biphasic co-flow step-emulsification, the emulsification regimes are determined by the critical capillary number, ${Ca}_{c}$. When the capillary number $Ca=12\mu q/\gamma wb$ is smaller than ${Ca}_{c}$, small droplets are formed at high frequency, while if $Ca>{Ca}_{c}$, large (“balloon”) droplets are generated at low frequency [2]. Here $\mu$ stands for the disperse phase viscosity, $q$ being the flow rate of disperse phase, $\gamma$ the surface tension between disperse and continuous phase, $w$ and $b$ are width and the height of the Hele-Shaw channel, respectively. For the disperse fluid with high viscosity, $Ca$ takes large values even at low flow rates, big “balloon” droplets are formed, as shown by the inset of Figure 5a in the main text. We find that the injection of air as the inner disperse phase forces the air and oil compound thread to break at the step, since air lowers the effective capillary number of the compound thread of the disperse phase [2]. We performed experiments to obtain the critical (minimal) inlet pressure $P_{c}$ of the air resulting in SE regime for formation of hollow-core double emulsions at high frequency. Figure S7 shows that the $P_{c}$ increases with increasing flow rate of the outer disperse oil. In addition, silicon oil with 3 different viscosity (69 mPa·s, 200 mPa·s and 500 mPa·s) are studied, that the $P_{c}$ for triggering air-oil compound thread to pinch at high frequency increases with increasing oil viscosity.

**
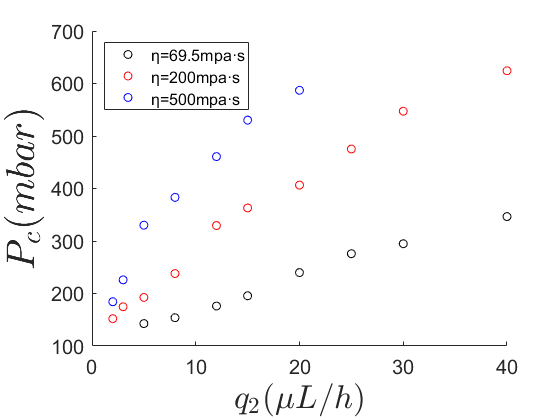
**

**Figure S8.** The critical (minimal) air pressure above which the SE regime occurs to generate hollow-core double emulsions, as a function of flow rate of the oil, for oils with 3 different viscosities.

**Supplementary Movie S1:** Formation of hollow-core double emulsions at the step. The inner disperse phase is air, the outer disperse phase is FC40 oil, and the continuous phase is SDS aqueous solution.

**Supplementary Movie S2:** The video showing stable generation of hollow-core double emulsions over 1h and 20min in a Hele-Shaw channel with depth $b=5 \mu m$. The inner disperse phase is air, the outer disperse phase is FC40 oil, and the continuous phase is SDS aqueous solution.

**Supplementary Movie S3:** The air core shrinks and disappears downstream in the reservoir, leaving single FC40 oil emulsion.

**Supplementary Movie S4:** Balloon-emulsification (BE) regime produces large hollow-core balloons, with ultrathin oil shell which dewets from the bubble surface, forming a mixture of oil droplets ranging from submicron to micron scale. The continuous aqueous phase, outer disperse phase, and inner disperse phase are: SDS aqueous solution, silicon oil with viscosity 69 mPa·s and air, respectively.

**References:**

[1] Merkel, T. C., Bondar, V. I. , Nagai, K., Freeman, B. D., Pinnau, I. Gas sorption, diffusion, and permeation in poly(dimethylsiloxane), J. Polymer Sci: PartB: Polymer. Phys. 38(3), 415–434 (2000).

[2] Li, Z., Leshansky, A. M., Metais, S., Pismen, L. M. & Tabeling, P. Step-emulsification in a microfluidic device. *Lab Chip*. **15**, 1023-1031 (2015).

[3] Ge, X. et al. Double emulsions with ultrathin shell by microfluidic step-emulsification.

*Lab Chip*. **21**, 1613-1622 (2021).
